# Evaluation of the Putative Duplicity Effect of Novel Nutraceuticals Using Physico-Chemical and Biological In Vitro Models

**DOI:** 10.3390/foods11111636

**Published:** 2022-06-01

**Authors:** Bianca-Maria Tihăuan, Mădălina Axinie (Bucos), Ioana-Cristina Marinaș, Ionela Avram, Anca-Cecilia Nicoară, Grațiela Grădișteanu-Pîrcălăbioru, Georgiana Dolete, Ana-Maria Ivanof, Tatiana Onisei, Angela Cășărică, Lucia Pîrvu

**Affiliations:** 1 Life, Environmental and Earth Sciences Division, Research Institute of the University of Bucharest, 050096 Bucharest, Romania; bianca.tihauan@sanimed.ro (B.-M.T.); ioana.cristina.marinas@gmail.com (I.-C.M.); gratiela.gradisteanu@icub.unibuc.ro (G.G.-P.); ana.ivanof@sanimed.ro (A.-M.I.); 2 Research & Development for Advanced Biotechnologies and Medical Devices, SC Sanimed International Impex SRL, 087040 Călugăreni, Romania; 3 Department of Science and Engineering of Oxide Materials and Nanomaterials, Faculty of Applied Chemistry and Materials Science, University Politehnica of Bucharest, 011061 Bucharest, Romania; dolete.georgiana@gmail.com; 4 Department of Genetics, Faculty of Biology, University of Bucharest, 030018 Bucharest, Romania; ionela24avram@yahoo.com; 5 Faculty of Pharmacy, University of Medicine and Pharmacy “Carol Davila”, 020021 Bucharest, Romania; anca.nicoara@yahoo.com; 6 Academy of Romanian Scientists, 010071 Bucharest, Romania; 7 National Research Center for Food Safety, University Politehnica of Bucharest, 060042 Bucharest, Romania; 8 National Institute of Research & Development for Food Bioresources—IBA Bucharest, 020323 Bucharest, Romania; tatianaonisei@yahoo.com; 9 National Institute for Chemical-Pharmaceutical Research and Development, 031282 Bucharest, Romania; angelacasarica@yahoo.com (A.C.); lucia.pirvu@yahoo.com (L.P.)

**Keywords:** nutraceuticals, prebiotics, diet, antioxidant, gut health

## Abstract

Nutraceuticals are experiencing a high-rise use nowadays, which is incomparable to a few years ago, due to a shift in consumers’ peculiarity tendencies regarding the selection of alternatives to Western medicine, potential immunity boosters, or gut-health promoters. Nutraceuticals’ compositions and actual effects should be proportional to their sought-after status, as they are perceived to be the middle ground between pharma rigor and naturally occurring actives. Therefore, the health benefits via nutrition, safe use, and reduction of potential harm should be the main focus for manufacturers. In this light, this study assess the nutritional profile (proteins, fats, fibers, caloric value, minerals) of a novel formulated nutraceutical, its physico-chemical properties, FTIR spectra, antioxidant activity, anthocyanins content, and potential hazards (heavy metals and microbiological contaminants), as well as its cytotoxicity, adherence, and invasion of bacteria on HT-29 cells, as well as its evaluation of beneficial effect, potential prebiotic value, and duplicity effect on gut microbiota in correlation with Regulation (EC) No 1924/2006. The results obtained indicate the growth stimulation of *Lb. rhamnosus* and the inhibitory effects of *E.coli*, *Ent. Faecalis* and *Lc. lactis*. The interaction between active compounds suggested a modulator effect of the intestinal microbiota by reducing the number of bacteria that adhere to epithelial cells or by inhibiting their growth.

## 1. Introduction

Diet–Gut Microbiome Interplay is a hot-topic relationship that, for the last decade, the scientific community has struggled to pinpoint. Although we still lack a clear picture of what a “normal microbiota” looks like, we do know that the digestive tract plays a vital role in the maintenance of host homeostasis and accomplishes many roles, including its normal development, digestion, vitamin synthesis, anti-infectious defense, immunity, and angiogenesis [1,2]. The host–microbe communication is influenced by age, diet, medication (mostly antibiotics), physical exercise, and associated diseases (such as diabetes or metabolic syndrome) [3]. Beyond the postnatal period, long-term dietary patterns have a strong influence on the composition of the gut microbiome. Therefore, given its importance for health, maintaining the balance of microbiota composition by different interventions, including diet-based ones, is critical [4]. A healthy diet shapes the gut microbiota composition, thereby influencing host–microbe interactions as well as homeostasis and disease processes [5]. 

Nowadays, consumers have gained more and more confidence in non-Western or natural, alternative products with beneficial effects on health, due to accessibility, few adverse reactions or contraindications, and the possibility of being combined with drug treatment (synergistic adjuvants—prebiotics). The development of microbiota-modulating products, such as prebiotics, is emerging to be of paramount importance, as it can be used as a supplement in food and nutraceutical applications. Nutraceuticals can be used as dietary supplements, as they are food ingredients or sourced from food products that, apart from their basic original nutritional value, provide extra benefits for the host including microbiota modulation [6]. For instance, dietary fibers alleviate several diseases by increasing the abundance and diversity of some microbes present in the gut [7]. Keeping in mind that, in the field of nutrition, plants and their products have significant importance not only for providing basic nutrients, but also for the prevention of various maladies. They indeed improve quality of life throughout the globe (plant-based traditional medicines have been in use since immemorial times). Consequently, dietary intervention with nutraceuticals and functional ingredients as dietary boosters has opened wide a window of opportunity for gut health nowadays.

The administration of nutraceuticals as well as of various supplements, such as fibers, antioxidant vitamins, omega-3 fatty acids, and herbs, has been employed to improve various diseases associated with dysbiosis [8]. In a recently published paper [9], we highlighted that research into the possible role of functional foods and nutraceuticals in mitigating immune function and actively sustain gut health is still in its infancy, especially in the Romanian market. 

Therefore, for this study we envisioned the evaluation of the effectiveness of a novel developed nutraceutical to be conducted in correlation with associated health claims approved by Regulation (EC) No 1924/2006 about nutrition and health claims made on foods, (EU) No 432/2012 made on vitamins and minerals, and EFSA regulations regarding the health claims of botanicals, keeping a strong focus on potential health benefits, duplicity effects, and the modulation of gut microbiota.

## 2. Materials and Methods

### 2.1. Nutritional Profiling of Novel Nutraceutic Formula

#### 2.1.1. Formulation Rationale

The novel nutraceutical (NN) formula developed was obtained using a pharmaceutical technology rationale [10]. All components were brought to the same dry powder form either by lyophilization or by atomization and at similar particle sizes by grinding them using a mill. Afterwards, all ingredients were combined using an electrically operated mixing apparatus; this process allowed us to obtain a homogenous powder with a high degree of dispersion. Metal sieves of different pore sizes were used to eliminate particles of larger dimensions. The nutraceutical formula is comprised of 48.5% hydrolyzed collagen, 25% egg white, 15% bee pollen, 5% *Prunus cerasus* (*sour cherries*) powder, 2.5% vitamin C, 2.5% electuary, 1% orange flavor, and 0.5% *Rumex patientia* (patience dock).

#### 2.1.2. Determination of Total Proteins

The total protein content was determined using the Kjeldahl method, using a conversion factor of total nitrogen to protein (6.25). Summarily, after digestion in concentrated sulfuric acid, the total organic nitrogen is converted to ammonium sulfate. Ammonia is formed and distilled into a boric acid solution under alkaline conditions. The borate anions formed are titrated with standardized sulfuric acid, from which is calculated the content of nitrogen, representing the amount of crude protein in the sample. The percentage of protein in a sample was calculated according to the Formula (1) [11]:

Protein nitrogen = (*b* − *a*)·*Ne*·1.4007·*f*/*Ws*·*n* (1)

were *b* is the volume of the 0.1 N sulfuric acid used in sample titration (mL), *a* is the volume of 0.1 N sulfuric acid used in blank titration, *Ne* is the expected normality of sulfuric acid solution (0.1N), *f* is the correction factor of the sulfuric acid normality obtained by using sodium carbonate solution, *Ws* is the weight (g) of sample, and *n* is the conversion factor of total nitrogen to protein (6.25).

#### 2.1.3. Determination of Extractible and Total Fats

Total fats were determined using the Soxhlet method [12]. Lipid determination was based on their solubility in organic solvents (i.e., acetone, ethyl ether, and petroleum ether) and was made using a Soxhlet extractor. The Soxhlet method is based on a continuous periodic extraction, using a well-determined solvent volume. The solvent used in the extraction goes through a closed cycle. The total product obtained after removing the solvents represents the gross fat. This method was applied to the analysis of all foodstuffs/food supplements being included.

#### 2.1.4. Determination of Ash Contents Insoluble in Hydrochloric Acid

Ash refers to the inorganic residue remaining after either the ignition or complete oxidation of organic matter in a sample. The inorganic residue consists mainly of mineral substances. Two major types of ashing procedure were commonly used, dry ashing and wet ashing. In the present work, the wet ashing method was used. This method is based on oxidizing organic matter using hydrochloric acid. Organic materials are incinerated at an elevated temperature (550 °C) in a muffle furnace, and inorganic matter (ash) remains. The ash content was measured using the weight of inorganic matter remaining.

#### 2.1.5. Determination of Moisture Content and Dry Matter

The moisture content and dry matter were determined using a Kern Moisture Analyzer DBS. This instrument works on the thermogravimetric principle. At the beginning of the measurement, the Moisture Analyzer determines the weight of the sample, the sample is heated with an inbuilt heating module, and the moisture vaporizes. During the drying process, the instrument continuously determines the weight of the sample and displays the reduction in moisture. When drying is finished, the results in terms of moisture content and/or dry weight of your sample are visible.

#### 2.1.6. Calorific Value

The carbohydrate content was determined using the difference method including fiber: Carbohydrates% = 100 − (moisture content% + crude protein% + fat% + fiber% + ash%). The gross energy values (kcal/100 g sample) were estimated using the Atwater’s conversion factor: 4 kcal/g for proteins, 4 kcal/g for carbohydrate, and 9 kcal/g for fat [13].

Food energy = (crude protein% × 4) + (fat% × 9) + (carbohydrates% × 4)

#### 2.1.7. Fourier-Transform Infrared (FT-IR) Spectroscopy

FT-IR spectroscopy is based upon the absorption of IR radiation by vibrational transition in the covalent bonds of biomolecules. The IR absorbance provides information about the samples’ contents depending on their structures, the molecular bonds, and their environments. FT-IR spectra were recorded using the Agilent Cary 630 FT-IR spectrophotometer in ATR mode, in the middle infrared region, the measurement range of which is between 4000–650 cm^−1^, the number of scans being 400, and the resolution is 4 cm^−1^. All the measurements were performed in triplicate.

#### 2.1.8. Determination of Micromineral Content

Concentrations of microminerals (Fe, Zn, Cu, Se, Mn) were determined using Agilent 8800 Triple Quadrupole ICP-MS (Agilent Technologies, Tokyo, Japan) in helium acquisition mode. The ICP-QQQ is equipped with an ASX500 autosampler, MicroMist concentric nebulizer, Peltier cooling spray-chamber (2 °C), 2.5 mm internal diameter torch, and nickel sampler and skimmer cones. The operating conditions of ICP-MS included 1550W RF power, 1 L/min carrier gas flow, 0.7 mL/min He flow, and a nebulizer pump set to 0.1 rps. Prior to analysis, the sample was digested in a microwave system (Ethos UP, Milestone Inc, Sorisole, Italy). A sample of precisely 0.5 g of powder was accurately weighed in a special TFM digestion vessel, and 8 mL nitric acid 65% (*v*/*v*) was added to the sample. Moreover, in order to remove excess carbon from the sample, we added an extra quantity of 1 mL hydrogen peroxide 30% (*v*/*v*). Finally, the vessels were screwed, positioned in the microwave system, and subjected to a preset digestion program (max. power: 1800 W; ramp time: 20 min, temperature 200 °C; hold time 15 min; and cooling: 10 min). Following digestion, the sample was cooled, transferred to a 50 mL polypropylene volumetric flask, and diluted to the mark with Milli-Q water. The digestion step was performed in triplicate for our sample, and the above-mentioned digestion program was also applied to a digestion blank and to 1 g certified reference material (SRM 1567b). Before measurements, the ICP-MS was tuned according to the manufacturer’s instructions and calibrated with five calibration standards in the following ranges: 0.1–2.5 µg/L for Hg, 0.1–5 µg/L for V, Cu, As, Cd, Pb, Rb, 0.5–25 µg/L for Mn, Zn, 5–100 µg/L for Al and Se and finally in the range 25–100 µg/L for Fe. The method was validated in terms of precision, accuracy, and linearity. Trace metal grade nitric acid 65% (*v*/*v*) and hydrogen peroxide 30% (*v*/*v*) were purchased from Merck. Ultrapure water with 18.2 MΩ cm resistivity was obtained by a Milli-Q^®^ system (Millipore, Bedford, MA, USA). Calibration standards were prepared from Multielement calibration standard 2A (p.n 8500-6940) and 2A-HG (p.n 8500-6940-HG) from Agilent Technologies, and reference material was procured from the National Institute of Standards and Technology (NIST, Gaithersburg, MG, USA), namely wheat flour SRM 1567b.

### 2.2. Assessment of the Physico-Chemical Properties

#### 2.2.1. Sample Preparation

An amount of 1 g of nutraceutical and its ingredients were weighed and brought into 50 mL of ethanol 50%. The extracts were obtained using the ultrasound-assisted extraction method involving frequencies ranging from 20 kHz to 2000 kHz for 30 min at room temperature. Then, the extracts were centrifuged for 10 min at 10,000× *g* rpm [14], filtered through a 0.45 µm porosity cellulose filter, and stored in the freezer until analysis

#### 2.2.2. Determination of Total Phenolic Content (TPC)

The TPC content was determined by the Folin–Ciocalteu method [15]. Briefly, an aliquot was mixed with 10 µL Folin–Ciocalteu reagent, 90 µL distilled water, and 100 µL of saturated sodium carbonate. The tubes were vortexed for 15 s and allowed to stand in the dark for 60 min for color development. Absorbance was then measured at 765 nm using a Multiskan™ Microplate Spectrophotometer (Thermo Fisher Scientific, Waltham, MA, USA). A standard curve was prepared by using different concentrations of gallic acid (R^2^ = 0.9983). The TPC content was expressed as milligram gallic acid equivalent/g nutraceutical (mg GAE/g).

#### 2.2.3. Determination of Flavonoid Content (TFC)

The total flavonoid content was assessed using the AlCl3 method described by Woisky and Salatino [16]. Briefly, 100 µL sample/standard solution was mixed with 100 µL 10% sodium acetate and 120 µL 2.5% AlCI3, the final volume being adjusted to 1 mL with 70% ethanol. The samples were incubated in the dark for 45 min, and the absorbance were measured at λ = 430 nm using a Multiskan™ Microplate Spectrophotometer (Thermo Fisher Scientific, Waltham, MA, USA). A standard curve was plotted using different concentrations (12.5–200 µg/mL) of quercetin (R^2^ = 0.9998). The total flavonoid content was expressed as mg quercetin equivalent/g of nutraceutical (mg QE/g).

#### 2.2.4. Total Anthocyanins Content

The method used was adapted as per A. Chiou et al. [17] and N. MohdMaidin et al. [18]. Briefly, the extract was mixed individually with pH 1.0 (HCl, 0.025 M) and pH 4.5 (sodium acetate buffer, 0.04 M) buffers in a 1: 5 (*v*:*v*) ratio and incubated at room temperature for 20 min. The absorbance of the test portions at both buffers was determined spectrophotometrically at wavelengths 520 nm and 700 nm using a Multiskan™ Microplate Spectrophotometer (Thermo Fisher Scientific, Waltham, MA, USA). The results of the anthocyanin pigments were expressed as malvidin-3-glucoside equivalents (mg ME/L) according to Formula (2):

(2) $$\mathrm{Total}\;\mathrm{Anthocyanins}\;(\mathrm{ME},\;\mathrm{mg}/L)=\frac{A\times MW\times DF\times 10^{3}}{\varepsilon \times l}$$

where *A* = (A520 nm − A700 nm)_pH1.0_ − (A520 nm − A700 nm)_pH4.5_; *MW*—molecular weight of malvidin-3-glucoside = 493.43 g/mol; *DF* = dilution factor; *l* = path length in cm; ε = 28,000 M extinction coefficient; and 10^3^ = factor for conversion from g to mg.

### 2.3. Assessment of Antioxidant Activity

#### 2.3.1. DPPH (2,2-Diphenyl-1-picrylhydrazyl) Assay

Performed according to the method described by G. Madhu et al. [19] with slight changes. The reaction mixture consisted of adding 100 µL of sample/standard and 100 µL of 0.3 mM DPPH radical solution to 70% ethanol. The absorbance was measured at λ = 517 nm after 30 min of incubation in the dark using a UV–VIS spectrophotometer. The concentrations used for the Trolox calibration curve were in the range of 5–80 µM (R^2^ = 0.9985).

#### 2.3.2. Cupric Ion Reducing Antioxidant Capacity Assay (CUPRAC)

The CUPRAC method is based on the reduction of a cupric complex, neocuproine, by antioxidants in copper form. A copper ion reduction was performed according to a method described by Celik et al. [20]: 60 µL of sample/standard solutions of different concentrations were mixed with 50 µL CuCl_2_ (10 mM), 50 µL neocuproine (7.5 mM), and 50 µL ammonium acetate buffer 1 M, pH = 7.00. After 30 min, the absorbance was measured at 450 nm. The stock Trolox solutions required for the calibration curve were 2 mM, and the working concentrations were between 0.24 and 2.0 mM (R^2^ = 0.9989). The results were expressed in mM Trolox/g of nutraceutical.

#### 2.3.3. Ferric Reducing Antioxidant Power Assay

The determination of the antioxidant capacity of the iron reduction was performed by the method described by Thaipong et al. [21]. The stock solutions included 300 mM acetate buffer, pH 3.6, 10 mM 2,4,6-tripyridyl-s-triazine (TPTZ) solution in 40 mM HCl, and 20 mM FeCl_3_ 6H2O solution in volume ratio 10:1:2, and then warming at 37 °C before using. After incubation, the absorbance was read at 593 nm. A 1 mM Trolox stock solution was used to plot the calibration curve, the concentration ranged between 30 and 250 µM Trolox (R^2^ = 0.9986). The results were expressed in mM Trolox/g of nutraceutical.

#### 2.3.4. Trolox Equivalent Antioxidant Capacity (TEAC) Assay

The assay was performed according to Re et al. [22] with a few modifications. A stable stock solution of ABTS+ was produced by mixing a solution of 7 mM ABTS in 2.45 mM potassium persulfate. Then, the mixture was left standing in the dark at room temperature for 12–16 h before use. An ABTS+ working solution was obtained by dilution with ethanol to an absorbance of around of 0.70. The reaction mixture consisted of 20 µL of sample/standard and 180 µL of ABTS+ working solution and was incubated for 30 min in the dark. The standard curve was linear between 20 and 200 µM Trolox (R^2^ = 0.9981). The results were expressed in mM Trolox/g of nutraceutical.

### 2.4. Hazard Analysis for Food Safety Assurance

#### 2.4.1. Determination of the Total Number of Aerobic Bacteria (Based on ISO 4833-1/2014 Standard)

The medium used for detecting aerobic bacteria was Plate Count Agar (PCA—Merck, Romania). On the surface of the medium, 100 µL of sample was dispersed on the entire surface using an L-shaped spreader. Both mesophilic and psychrotrophic bacteria can be detected by incubation at 30 °C for three days. After the incubation period, all the colonies from the plates were numbered. All samples were processed in triplicate. Interpretation of results was performed using the following Formula (3):

(3) $$N=\frac{\sum^{\;}C}{\left(n_{1}+0.1n_{2}\right)\times \;d}$$

where: N= total number of aerobic bacteria; ΣC = sum of colonies counted in all retained plates; n_1_ = number of plates retained at first dilution; n_2_ = number of plates retained at the second dilution; and d = dilution from which the first counts were made.

#### 2.4.2. Determination of the Total Number of Fungi (Based on ISO 21527-2:2009 Standard)

The medium used for the determination of fungi was Dichloran glycerol Agar with chloramphenicol (DG 18, Merck, Romania) and the samples were incubated at 25 °C for seven days. As various molds and yeasts grow on this medium, DG18 is recommended for selective isolation of xerophilic molds from food samples. It is a medium suitable for the enumeration of xerophilic fungi from low-moisture foods. On the surface of the medium, 100 µL of sample was dispersed on the entire surface using an L-shaped spreader. After the incubation period, the colonies were counted and analyzed according to the formula 3 previously mentioned. All samples were processed in triplicate.

For Section 2.4.1 and Section 2.4.2, the water activity (WA) was determined using the formula WA = relative humidity (%)/100. Most foods have a water activity above 0.95, which will provide sufficient moisture to support the growth of bacteria, yeast, and mold.

#### 2.4.3. Determination of Heavy Metals

Hazardous toxic metals (As, Cd, Pb, Hg) and other potential harmful metals, if exposed to high doses, such as V, Al and Rb, were determined using an Agilent 8800 Triple Quadrupole ICP-MS (Agilent Technologies, Tokyo, Japan) in helium acquisition mode. The ICP-QQQ is equipped with an ASX500 autosampler, MicroMist concentric nebulizer, Peltier cooling spray-chamber (2 °C), 2.5 mm internal diameter torch, and nickel sampler and skimmer cones. The operating conditions of the ICP-MS included 1550 W RF power, 1 L/min carrier gas flow, 0.7 mL/min He flow, and a nebulizer pump set to 0.1 rps. The sample was processed as described in Section 2.1. The same reagents and calibration materials as described in Section 2.1 were used.

### 2.5. Evaluation of Putative Duplicity Effect on Probiotic and Pathogenic Bacteria

An aqueous extract was obtained from the NN powder (400 mg powder dissolved in 1 mL Phosphate Buffer Solution—PBS) by cold ultrasound-assisted extraction. The extract thus obtained was centrifuged for 15 min at 8000× *g* rpm to remove insoluble material and then sterilized by filtration through 0.2 µm cellulose membranes. Microbial susceptibility was assessed according to CLSI 2019 M100 [23]. After 18–24 h of incubation at 36 ± 2 °C, serial dilutions were made from the first sample, and the number of viable cells was assessed by calculating CFU/mL. In this study, various probiotic strains were included, such as *Lactococcus* (Lc.) *lactis* DSM 20729, *Enterococcus* (E.) *faecalis* ATCC 19433, *Lc. lactis* CMGBL27 (isolated from human faeces), *Lacticaseibacillus* (L.) *rhamnosus* ATCC 9595, *L. rhamnosus* CMGB 38 (isolated from human faeces) *Saccharomyces* (S.) *cerevisiae* var. *boulardii* CMGB102, and gut pathogenic strains *Enterococcus faecalis* CMGB 35 (isolated from human faeces) *Enterobacter* (Ent.) *aerogenes* IC 13488, *Salmonella* (S.) *enterica* subsp. *enterica* ATCC 14028, *Escherichia* (E.) *coli* ATCC 25922, both types of standardized and clinical origin. All samples were processed in triplicate.

### 2.6. Cytotoxicity

HT-29 human epithelial cells (colon adenocarcinoma) (ECACC—European Collection of Authenticated Cell Cultures) were selected as the model for the cytotoxicity assessment of the NN sample (prepared as mentioned in Section 2.5). The samples were analyzed from a starting concentration of 400 mg/mL and serially diluted in order to obtain the following concentrations: 200 mg/mL, 100 mg/mL, and 50 mg/mL. PBS was used as negative control. HT-29 cells were cultivated in a RPMI-1640 culture medium (Sigma-Aldrich, St. Louis, MO, USA) supplemented with 2 mM Glutamine (Sigma-Aldrich), 10% heat inactivated Fetal Bovine Serum (FBS) (Sigma-Aldrich) and 1% Pen/Strep (penicillin /streptomycin solution, 50 µg/mL—Sigma-Aldrich) for 24 h at 37 °C, 95% humidity with 5% CO_2_. After 24 h, cells were washed with PBS (Phosphate Buffered Solution—Sigma-Aldrich), harvested using trypsin (Sigma-Aldrich) and counted using Trypan Blue (Sigma-Aldrich) and a hemocytometer. The seeding density for the MTT and LDH assays was optimized at 4 × 10^5^. Cells seeded at 4 × 10^5^ density in a clear 96 well cell culture plate were treated with NN samples and controls and incubated for 24 h at 37 °C, 95% humidity with 5% CO_2_. After 24 h of exposure to the tested compounds, the cells were incubated for 4 h with MTT reagent (Sigma, USA) at 37 °C, 95% humidity with 5% CO_2_. After incubation, the cells were treated with MTT solvent (Sigma, USA) for 15 min at room temperature. The absorbance was measured using a spectrophotometric microplate reader (Synergy™ HTX Multi-Mode Microplate Reader, Biotek, Winooski, VT, USA) at OD = 570 nm. LDH activity was measured using a spectrophotometric microplate reader at 492 nm with a 600 nm wavelength reference with the LDH Cytotoxicity Detection Kit (Roche).

### 2.7. Adherence and Invasion of Bacteria on HT-29 Cells

A model of intestinal mucosa was represented by HT-29 human epithelial cells cultivated until 80% confluence in the conditions described in Section 2.6. The microbial strains used for the assessment of adherence were selected based on the susceptibility test results assessed in Section 2.5. In a 6-well plate, the seeded cells were washed vigorously two times using warm PBS, then inoculated with 0.9 mL of microbial suspensions of 0.5 McFarland of *S. typhimurium* ATCC 14028, *E. coli* ATCC 25922, *Lc. lactis* DSM 20729, *E. faecalis* ATCC 19433, and *L. rhamnosus* ATCC, and 100 µL of NN sample was added. The plates were then incubated for 2 h at 37 °C. After 2 h, the cells were washed again three times with warm PBS, fixed using methanol (Merck), dried down, and stained using Gram’s method [24]. After staining, the plates were dried and examined by optic microscopy (Axiolab 5, Zeiss, Munich, Germany) using the immersion objective in order to evaluate the adherence indexes and patterns.

For the invasion assay, the culture medium of the HT-29 monolayers was removed and replaced with a warm RPMI medium with 10% heat inactivated FSB. The 10 µL suspensions of 0.5 McFarland of *Campylobacter* (C.) *jejuni* NCTC 81-176 were added over 990 µL of culture media and on top 50 µL of NN sample. Plates were incubated for 15 h at 37 °C, 95% humidity with 5% CO_2_. Cells treated with PBS were used as negative control. After incubation, cells were washed three times with PBS, and half of the cells seeded in the 24-well plate were treated with 100 µL of Triton X 0.1%, incubated 15 min at 37 °C, and then plated on Mueller Hinton agar for CFU count. The other half was treated with gentamicin-sulfate 50 µg/mL, incubated for 2 h at 37 °C, then washed six times with PBS, then 100 µL of Triton X 0.1% was added to the sample, which was incubated 15 min at 37 °C and then plated on Mueller Hinton agar for CFU count.

### 2.8. Statistical Analysis

For biological assessments, statistical analyses were performed using a GraphPad Prism 9 (San Diego, CA, USA). Data were analyzed using the two-way ANOVA test and *t* test. The level of significance was set to *p* < 0.05. For the physico-chemical assessments, data were expressed as means ± SD determined by triplicate analysis. The statistical analysis was conducted using a GraphPad Prism 9 (San Diego, CA, USA). Data were analyzed using an unpaired *t*-test with Bonferroni–Dunn’s multiple comparisons of the antioxidant activity and chemical content (phenols, flavonoids, anthocyanins) of the final product and the cumulative effect of the ingredients. The level of significance was set to *p* < 0.05.

## 3. Results

According to Regulation (EC) No 1924/2006 nutritional profiling of novel formulated dietary supplements/nutraceuticals constitutes the starting point in pinpointing quality parameters, as well as drawing conclusions for eventual nutritional benefits.

Therefore, we evaluated the total protein content (%), total lipidic content (%), ash insoluble in 10% HCl (%), moisture content (%), and dry matter (%) for all ingredients that were added into the NN formula, as well as their fingerprints in the final product (Table 1).

**Figure 1:**
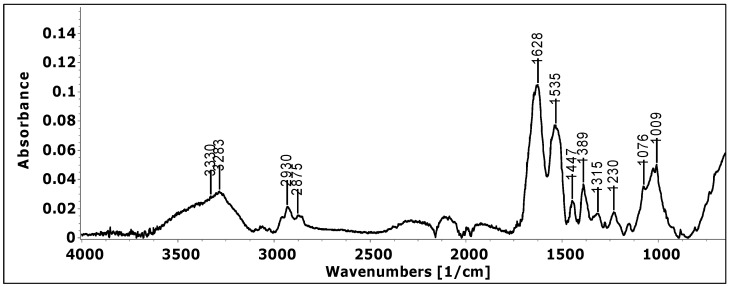
FT-IR spectra of the final product (NN sample).

According to previously reported data, the absorption spectra of high-protein products show specific peaks, i.e., Amide I (about 1628 cm^−1^) and Amide II (about 1535 cm^−1^) bands. The former arises primarily from the C=O stretching vibration, and the latter is attributed to the N-H bending and C-N stretching vibrations of the peptide backbone. The band with the maximum absorption at about 3283 cm^−1^ was assigned to Amine A. The absorption band at 3000–2875 cm^−1^ corresponds to the C-H stretching vibration, while the low intensity band at about 1076 cm^−1^ is due to the C-O stretching (Figure 1).

Peaks specific for phenolic compounds have also been identified. So, the bands 1233 cm^−1^ are a function of the protonation of phenolic acids and are assigned to the stretching vibration of C-OH in the carboxylic group [25]. The peak at 1390 cm^−1^ is characteristic of COO stretching [26]. Aromatic C-C extending to ~1520 (peak overlapping with the specific Amide II peak) and ~1447 cm^−1^ is related to phenolic compounds [27]. The band at ≈2900 cm^−1^ corresponds to the C-H stretching vibration that reflects the organic content in general [28]. Several peaks located in the region of 1070–1150 cm^−1^ are mainly attributed to stretching vibrations of C-O and C-C [29]. The absorptions at 1230 cm^−1^ have been assigned to phenol hydroxyl group [30]. The stretching of ether linkage, C-O-C, is indicated by the absorption at 1009 cm^−1^ [31].

The high protein content (80.97%) of the final formulation is a result of using high proportions of hydrolyzed collagen (90.30%) and egg white (82.05%), two important sources of high-quality proteins, which might contribute considerably to the recommended human daily protein allowance. Taking into account that the general recommendation for daily protein requirements is a minimum of 0.8 g protein/kg body weight/day, the protein intake provided by the new nutraceutical formula represents 27% of the recommended daily dose for adults.

Due to the fact that ingredients with a high content of good-quality protein were used, the amount of ash insoluble in 10% HCl obtained for the final supplement formulation is very low (0.27%). The water activity measurement is also described, since it parallels the measurement of the total moisture as an important stability and quality factor. The dry matter that remains after moisture removal is commonly referred to as total solids; this was 7.37% for the final formulation. The moisture content of suppliants is significant for their shelf life, with better storage stability maintained by lower moisture contents of the supplements [32].

The fiber content for the new nutraceutical formula (2.1%) was calculated based on data provided by the producer for the two fiber-containing ingredients: pollen (13%) and cherry powder (3%). The carbohydrate content, determined by difference, was 8.42%, while the energy values are 365 kcal/100 g.

The assay used for the determination of microminerals, hazardous toxic metals, and other potential harmful metals showed good linearity for all elements in the selected ranges with correlation coefficients greater than 0.999.

The concentrations of the metals in the sample are showed in Table 2 and are expressed as the mean value of the triplicate analysis, followed by the standard deviation. Toxic metals such as Cd, Pb, and Hg were compared with the maximum levels according to Commission Regulation (EC) No. 1881/2006. Lead, cadmium, and mercury are the only metals that are specifically regulated for dietary supplements, with maximum levels of 3 mg/kg for Pb, 1 mg/kg for Cd, and 0.1 mg/kg for Hg. Arsenic, on the other hand, is not specifically regulated for dietary supplements, but we took in consideration the smallest value that is specified in (EC) No. 1881/2006, respectively, 0.1 mg/kg [33].

As we can see from Table 2, our sample does not harbor alarming values for heavy metals. Although arsenic, cadmium, and lead are present in the samples in concentrations of 0.0406 µg/g, 0.0266 µg/g, and 0.0534 µg/g, respectively, these values do not exceed the maximum regulated limits, while mercury has not been detected in any form, being below the instrumental detection limit.

As for the rest of the analytes, microminerals are not present in quantities that might exceed the daily population reference intakes (PRI) established by the EFSA [34]. The requirements for these microminerals vary from grams per day, for elements such as Na or K, to milligrams per day, for elements such as Fe and Zn, and even to micrograms per gram (Cu, Se, Mn). The most abundant species found in our sample are iron, manganese and aluminum, with concentrations of approximately 23 µg/g, 17 µg/g and 17 µg/g, followed by zinc and rubidium with a concentration of ~8 µg/g and 6 µg/g.

All ingredients in terms of phenolic, flavonoid, and anthocyanin content were analyzed. Among the ingredients used in the formulation of the food supplement, pollen had the highest polyphenols and flavonoids. A higher value for Vitamin C was actually generated by the reduced capacity of the Folin-Ciocalteu reagent (Table 3). The present method is not suitable for the determination of the total phenolic content unless interfering substances are considered or removed. Moreover, the application of this method for the determination of the antioxidant capacity of food samples is proposed for the evaluation of the contribution from phenolic and other reducing substances [35]. By evaluating the percentage of phenolic and flavonoid compounds quantified in the final formulation compared to the cumulative value of all ingredients from formulation, it was observed that some of the compounds are denatured by the technological process. No significant changes were observed in the case of anthocyanins. The higher values of the electuary and orange flavors for phenolic and flavonoids content were given by the precipitation with alcoholic media. For the final quantification, we took account of these aspects.

In the case of antioxidant activity, a significant difference was observed between the cumulative effects of the ingredients compared with the finished product for the TEAC and CUPRAC variants (Table 4), but the synergistic effect between the components was relatively low. In the case of DPPH and FRAP, even if the value of *p* < 0.05, according to the model these are not significant due to the high values of the standard deviations. So, we consider that the ingredients were not antagonistic, but the effect for TEAC and CUPRAC was synergistic, while for DPPH and FRAP it was additive [36].

The evaluation of potential microbial hazards results obtained from the microbiological analysis of the NN powder revealed absence of contamination. The total number of aerobic bacteria and the total amount of yeast and mold were expressed in CFU/g. The recorded results indicate for both assays <10 CFU/g (Table 5), implying that the sample presents no microbiological risk, therefore the quality of the finished products was not affected.

Both mesophilic aerobic bacteria and fungi are common spoilage microorganisms that contaminate the technological flow under conditions of inadequate hygiene. In order to prevent the contamination of finished foodstuffs with microorganisms, a rigorous inspection of the raw materials, working materials, staff hands, the environment in which the product is produced, and the processing method must be performed. These are potentially contaminating elements of the technological flow.

Water activity is the most important parameter of water in terms of food safety. The value of water activity (aw) for food is an essential criterion for the microbiological control of products. Water activity is defined as follows: when a hygroscopic material is placed in a closed chamber, a balance will be achieved between the material and the air above it. Relative humidity, which occurs at a constant air temperature, corresponds to the value of water activity multiplied by 100 (aw = relative humidity (%)/100). A water activity above 0.95 will provide sufficient moisture to support the growth of bacteria, yeast, and mold. The samples tested showed a low value of water activity.

**Figure 2:**
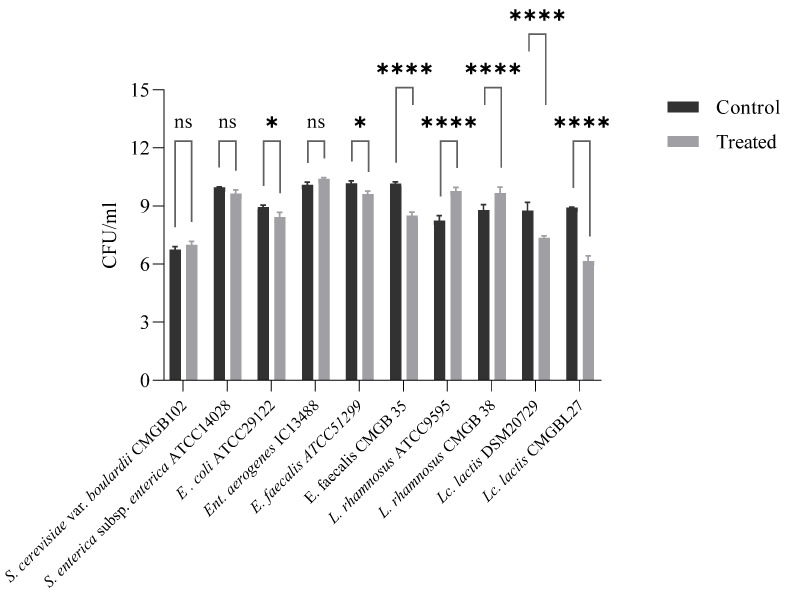
Bacterial cell viability in the presence of NN product; *p* value *, **** < 0.05; ns = *p* value > 0.05 (non-significant statistically).

The evaluation of the supposed putative duplicity effect of NN with a prebiotic role was performed by addressing the dose-response on the viability of both the probiotic and pathogenic bacteria that are specific to gut microbiota. The results obtained indicate a promotion of viability for probiotic bacteria and an inhibitory effect on pathogenic bacteria, which reinforce our hypothesis that prebiotic formulas have a duplicity effect (Figure 2). This finding wisely emphasizes the concept of Duplibiotics, which was recently introduced into the scientific literature and mentioned by Rodríguez-Daza M.C. et.al. [37].

*Lb. rhamnosus* strains are present in various probiotic products and are associated with multiple beneficial effects on human health. The active compounds present in the NN product stimulate 10 times the growth of *Lb. rhamnosus* strains. The new product has inhibitory activity against *E. coli*, *Ent. Faecalis*, and *Lc. Lactis*; the number of bacterial cells is being reduced by the bioactive compounds present in the NN product. In contrast, nutraceuticals have no effect on *Sal. Typhimurium*, *Ent. Aerogenes*, and *S. boulardii* growth. Our results showed that NN exerts a selective effect on the growth of the bacterial strains present in the intestine.

In our study, we selected colorimetric assays for cytotoxicity assessment. The MTT assay is a very popular and widely used colorimetric assay in the in vitro evaluation of cytotoxicity [38], as it provides beneficial aspects such as rapidity, reliability, and significant knowledge regarding the metabolic activity of eukaryotic cells. The LDH assay provides information about cellular damage (lysis of cells membranes) after cell treatment; it complements the MTT assay in drawing conclusions about the potential mechanism of action.

**Figure 3:**
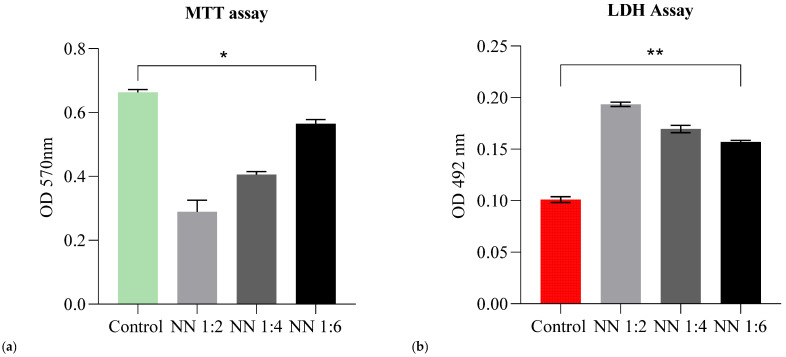
Graphical representation of MTT (**a**) and LDH (**b**) assays results for NN sample of 200 mg/mL, 100 mg/mL and 50 mg/mL concentrations. Control is represented by HT-29 cells in culture media with PBS (NN solvent). For MTT *p* value * = 0.0103 (highly significant statistically) and for LDH *p* value ** = 0.0042 (highly significant statistically).

The NN sample was analyzed at three different concentrations of 200 mg/mL (NN 1:2), 100 mg/Ml (NN 1:4) and 50 mg/Ml (NN 1:6). The results obtained revealed that high concentrations of NN (200 mg/mL) induce a significant cellular viability reduction (Figure 3a) (up to 56% viability loss), followed by the 100 mg/mL concentration (with 39% viability loss). The greatest percentage of viability was obtained for the 50 mg/mL concentration (with only 15% viability loss). In this case, at 50 mg/mL the NN powder had a beneficial effect on HT-29 cells and acted as a nutritive substrate (viability rates similar to control—untreated cells).

Even though the tested formulations affected the cell proliferation at 200 mg/mL and 100 mg/mL (as indicated by the MTT assay), they did not exhibit cytotoxic effects on the intestinal cells at 50 mg/mL (viability rates being similar to control cells). Moreover, NN 1:6 showed LDH values similar to the control (unstimulated cells).

Bacterial adherence to monolayers of HT-29 cells in the presence of NN sample extract resulted in index values between 82% and 92%. The most significant inhibitory effect has been noticed for the *Escherichia coli* ATCC 25922 strain (Table 6).

The inhibition of bacterial adhesion, invasion, and intracellular survival significantly limits the pathogenicity of microbial agents and provides a beneficial background for the prevention and control of associated infections [39,40]. For this assay, we selected a *Campylobacter jejuni* NCTC 81-176 strain, a Gram-negative spirally curved microaerophilic bacterium that is recognized as a significant cause of human enteritis (34) (campylobacteriosis), being one of the most widespread infectious diseases of the last century. The incidence and prevalence of campylobacteriosis have increased in both developed and developing countries over the last 10 years [41]. Therefore, we tested the effect of NN at a concentration of 200 mg/mL against bacterial adhesion and invasion.

**Figure 4:**
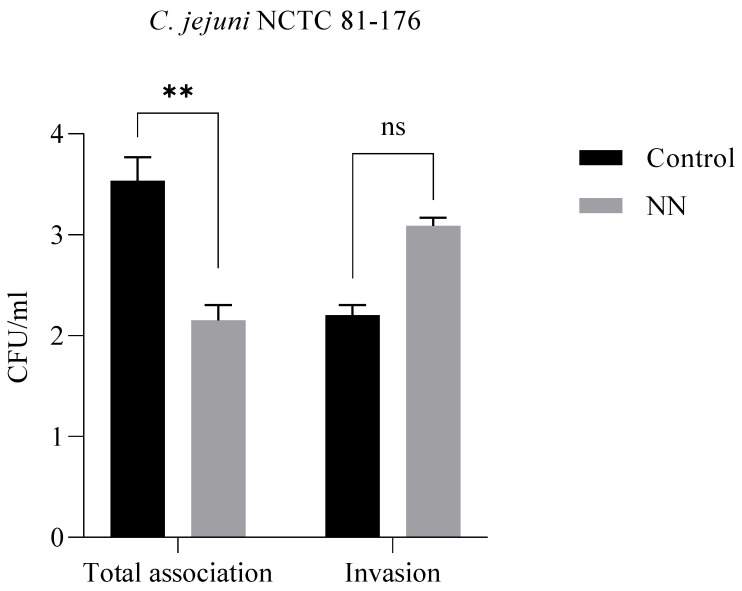
*C jejuni* NCTC 81-176 adhesion and invasion; *p* value ** = 0.0047; ns = *p* value > 0.05 (non-significant statistically).

The results obtained (Figure 4) indicate that the total number of bacterial cells adhering to the epithelial cells is significantly lower in the sample in which the NN product was added, while the new product had no effect on the *C jejuni* invasion.

## 4. Discussion

We started this study by asking ourselves if nutraceuticals are a gut friend or a foe. In trials for deciphering this conundrum, we came across various controversies over a specific definition and set of regulations to define a nutraceutical compound. We understand now that the term is recognized and accepted for use for health-enhancing products that improve the mental and physical activities of the body [42,43]. Straightforwardly, a nutraceutical product can be defined as “a food or part of a food that provides benefits to health in addition to its nutritional content” [44].

The general health status of a human host presents an interdependence alliance with the gut microbial ecosystem. The microbiota mediate interactions between the internal and external environments in the gut, forming an intimate partnership among intestinal epithelial cells and dietary nutrients. Scientific findings support the correlation between long-term dietary patterns and their influence on the composition of gut microbes [45]. Not surprisingly, diet and gut microbes are two critical factors linked with the pathogenesis of gastrointestinal diseases and systemic conditions such as metabolic and cardiac disorders [45]. Therefore, promoting the use of nutraceuticals is a tempting intervention in terms of how to manipulate diet to promote a healthy microbiota.

Dietary intervention via nutraceuticals opened wide a window of opportunity for gut health. Given its importance for health, maintaining the balance of microbiota composition, by different interventions including diet-based ones, is critical [46,47]. A healthy diet shapes the gut microbiota composition, thereby influencing host–microbe interactions as well as homeostasis and disease processes [5].

According to Regulation (EC) No 1924/2006 on nutrition and health claims made on foods, (EU) No 432/2012 on vitamins and minerals, and EFSA regulations regarding health, claims of botanicals, ingredients used in our NN formula have the following recognized functions: *Prunus emarginata*—antioxidant activity, protects the general health by antioxidant activity ID4507; bee pollen—helps to improve immunity ID3136, enhances appetite ID3135, supports and promotes good functioning of the hearts, blood vessels and a balanced level of blood lipids ID4648, is a natural source of compounds with antioxidative action that helps maintain the optimum antioxidant status of the body ID4659; vitamin C—contributes to the normal function of the immune system ID4321, contributes to the protection of cells from oxidative stress ID 3331, contributes to the reduction of tiredness and fatigue ID2622, contributes to normal energy-yielding metabolism ID3196. As for the product per se, the following claims align with (EC) No 1924/2006: good source of proteins and fibers, sugar-free, and saturated fat-free.

The nutritional profiling of the final formulation indicates a high protein content (80.97%) owed to the main ingredient of the formula, hydrolyzed collagen, and to the egg whites. Collagen plays a supportive role in rebuilding and strengthening the lining of our digestive tract as it contains the amino acids—particularly glycine and glutamine—that are essential for its repair [48]. Furthermore, egg whites are a good source of protein and a reasonable option for individuals affected by diabetes, high cholesterol, or cardiovascular disease [49]. Due to several formulation tryouts, we succeeded in obtaining a low level of fats; out of all ingredients, the highest-ranking one was the bee pollen, which more than reasonably provided important health benefits that counterbalanced the final product. The calorific value of the final product is 365 kcal/100 g and 73 kcal for a single-serving portion.

In terms of phenolic and flavonoid content, bee pollen presents the highest amount; these results effectively support its antioxidant potency and correlate with other authors findings [50]. According to Oroian et al. (2020) [51], among the phenolic compounds, the majority compounds are 5-O-caffeoylquinic acids and caffeic acid from the phenolic acids group, quercetin 3-O-galactoside and isorhamnetin 3-O-glucoside from the flavonols group, and luteolin and apigenin from the flavones group. It is well known that the chemical composition of pollen differs significantly depending on the plant species, whether it is uniflora or multiflora, the climatic-geographical conditions [52], and its processing methods and storage environment [53,54]. Due to the fact that a higher value for Vitamin C was generated by the reduced capacity of the Folin-Ciocalteu reagent, the results obtained for it should not be taken into account for the total phenolic and flavonoid content. Moreover, an influence of the technological process selected for product formulation was observed; however, it did not affect the potency of ingredients in a major way, but, nevertheless, it should be considered for further improvement. The anthocyanin content obtained for sour cherries was relatively high (165.95 µg/g); their antioxidant activity values are similar to other reported results [55], mainly due to the presence of polyphenol but also to the anthocyanin fractions as well.

The evaluation of the antioxidant activity of the final product indicates that the combination of ingredients does not lead to antagonistic effects, the effect for TEAC and CUPRAC was synergistic, while for DPPH and FRAP it was additive. The DPPH values are also influenced by organic solvent-soluble antioxidant compounds [56], while FRAP values are strongly influenced by the pH of the reaction medium, some antioxidant compounds may undergo a modified antioxidant effect [57]. The FRAP method has been used successfully on a large scale to determine the antioxidant activity of anthocyanins in various matrices, so the highest value obtained for sour cherries was that obtained using the FRAP method [58]. In the case of vitamin C, the results obtained correlate with the mechanism proposed by Liu et al. [59]; higher values being given by the methods that have as HAT mechanism of action (TEAC and DPPH) and lower values for the methods, SET ones (FRAP and CUPRAC) [60]. Among the ingredients used, the antioxidant activity is given mainly by Vitamin C, pollen, and sour cherries.

The potential hazards of microbiological and chemical origin were evaluated. The results obtained for the total number of aerobic bacteria and total number of yeasts and fungi were in compliance with values indicated as safe for consumption by the ISO 4833-1/2014 and ISO 21527-2:2009 standards.

As for heavy metal content, our sample does not show alarming values. The values obtained for arsenic, cadmium, and lead do not exceed the maximum regulated limits, while mercury has not been detected, as it was below the instrumental detection limit. Iron, manganese, and aluminum were the most abundant microminerals determined in the final product, but their values did not exceed the daily population reference intakes (PRI) established by the EFSA [34].

The putative duplicity effect of a NN with a prebiotic role was addressed by evaluating the dose-response on probiotic and pathogenic bacteria that are specific and important to the gut ecosystem. A 10× stimulatory response was obtained for the *Lb. rhamnosus* strains. This species is appraised for its resistance to acid and bile, good growth characteristics, and adhesion capacity to the intestinal epithelial layer, antimicrobial activity factors that contribute to its probiotic status [61]. Since dietary supplementation with probiotics in various health-affecting conditions (infections, diarrhea, etc.) is not necessarily a fit-all solution, this result is of significant importance, because it promotes the growth of a self-bacterial population. Moreover, our product presented an inhibitory effect against *E. coli, Ent. Faecalis*, and *Lc. lactis* strains, with the reduction in bacterial cells number being significant. In contrast, on *Sal. Typhimurium, Ent aerogenes*, and *S. boulardii* strains, the nutraceutical presented no effect on growth. Although the number of tested strains was limited, the selective effect which was observed reinforces its supposed duplicity effect and encourages us to extend the study to more clinically isolated strains in order to confirm the hypothesis.

Evaluation of the in vitro cellular response plays an important role in safety assessments and assurances. Both the EFSA and FDA regulate finished dietary supplement products (such as nutraceuticals/prebiotics) in order to ensure safe consumption before the product is placed on the market. Compliance with the aforementioned regulations is mandatory. For this study, a safety assessment was performed on a cell line representative for the human gut (HT-29). We addressed the viability, proliferation, and cytotoxicity using MTT and LDH assays. The obtained results indicated viability rates similar to the control (untreated cells), especially for the NN concentration of 50 mg/mL; this result may be due to the high percentage of collagen hydrolysate (48.5%), which is usually associated with beneficial effects on cell proliferation by providing a nutritive substrate [62,63,64].

For an assessment of the influence on the inhibition of bacterial adhesion and invasion, we selected as a model a strain of *Campylobacter jejuni* NCTC 81-176. Its structural characteristics and especially the binding proteins CadF and FlpA facilitate the invasion and intracellular survival [65]. Moreover, due to its capacity to cause one of the most widespread infectious diseases of the last century, we considered it more than relevant for this assay. While no effect on invasion capacity was observed, the bacterial cells adhering to the epithelial cells were significantly lower in the sample in which the NN product was added.

As we walked through every testing phase, we demonstrated that our product shows good prebiotic effects by providing a balanced nutritional profile by promoting a duplicity effect through the growth enhancement of probiotic bacteria and the growth inhibition of pathogenic strains, and by interfering with the adherence mechanisms of *C. jejuni,* therefore exhibiting an overall health beneficial impact. Keeping in mind that various factors including dysbiosis play a critical role in pathologies such as type 2 diabetes and metabolic syndrome, microbiome modulation via prebiotics is considered a therapeutic approach [66,67] targeted for the alleviation of metabolic outcomes. For the general population (healthy subjects), this type of prebiotic/nutraceutical acts as an adjuvant, boosting immunity by its antioxidant potential and contributes to the reduction of tiredness and fatigue and to normal energy-yielding metabolism.

## 5. Conclusions

One of the objectives of this study was to evaluate a novel formulated nutraceutical product with scientific and legislative rigor in order to bring it as close to a marketable prototype as possible. Some improvements are envisioned, especially for the stability of the product, as well as its antioxidant potential. As for its prebiotic effect, we obtained great results for the growth stimulation of *Lb. rhamnosus* as well as inhibitory effects of *E. coli*, *Ent. Faecalis*, and *Lc. lactis*. The interaction of the active compounds present in the final product NN showed that it could be used as a modulator of intestinal microbiota. The product also works through multiple mechanisms, such as reducing the number of bacteria that adhere to epithelial cells or by inhibiting the growth of some pathogens. Therefore, the intended use of the novel formulated nutraceutical is as a prebiotic and immunity booster. It also presents a good candidate for further in vivo studies.

## Figures and Tables

**Table 1:** Nutritional status and physico-chemical properties of ingredients and final formulation.

Samples	Total Proteins %	Total Lipids %	Ash Insoluble in HCl %	Moisture Content %	Dry Matter %	Fiber %
**Hydrolyzed Collagen**	90.30	-	0.28	7.74	92.26	-
**Egg white**	82.05	0.37	0.54	9.85	90.15	-
**Pollen**	24.75	5.16	0.20	7.24	92.76	13 *
**Cherries powder**	0.93	0.39	0.14	4.43	95.57	3 *
**Orange flavor**	-	-	0.02	6.80	93.20	-
**Vitamin C**	-	-	0.08	0.80	99.20	-
**Patience dock**	-	-	0.02	0.42	99.58	-
**Electuary**	-	-	0.01	0.80	99.20	-
**Final product SAN**	80.97	0.87	0.27	7.37	92.63	2.1 **

* value provided by the producer; ** value calculated from the data provided by the producer.

**Table 2:** Results obtained for selected elements reported as mean values ± SD, *n* = 3.

Al	V	Mn	Fe	Cu	Zn
(µg/g)	(µg/g)	(µg/g)	(µg/g)	(µg/g)	(µg/g)
16.69 ± 0.19	1.13 ± 0.02	17.17 ± 1.27	22.63 ± 0.37	1.88 ± 0.05	8.41 ± 0.23
**As**	**Se**	**Rb**	**Cd**	**Hg**	**Pb**
(µg/g)	(µg/g)	(µg/g)	(µg/g)	(µg/g)	(µg/g)
0.0406 ± 0.0009	0.4744 ± 0.0063	5.96 ± 0.06	0.0266 ± 0.0010	n.d	0.0534 ± 0.0176

**Table 3:** Chemical content of phenolics, flavonoids, and anthocyanins for ingredients and final product.

Ingredients	Total Phenolic Content (mg GAE/g)	Total Flavonoid Content (mg QE/g)	Total Anthocyanins Content (µg ME/g)
**Vitamin C**	1137.85 ± 0.91	-	-
**Patience dock**	0.03 ± 0.00	-	-
**Pollen**	98.15 ± 2.70	33.88 ± 0.19	-
**Hydrolysis collagen**	1.62 ± 0.10	-	-
**Egg white**	0.17 ± 0.02	0.05 ± 0.00	-
**Sour cherries**	1.28 ± 0.11	0.51 ± 0.03	163.95 ± 2.75
**Orange flavor**	0.56 ± 0.01	1.38 ± 0.12	-
**Electuary**	3.03 ± 0.04	4.71 ± 0.09	-
**Sum of the compounds to the mass ratio of ingredients**	44.14 ± 0.12	5.25 ± 0.00	8.10 ± 0.27
**Final product**	35.95 ± 0.91	4.52 ± 0.08	8.27 ± 0.18
***p*** **-value**	<0.001	<0.0001	>0.05
**Percentages of compounds in final formulation (%)**	81.44	86.07	102.10

**Table 4:** Antioxidant activity of ingredients and final product.

Ingredients	TEAC (mg Trolox/g)	DPPH (mg Trolox/g)	FRAP (mg Trolox/g)	CUPRAC (mg Trolox/g)
**Vitamin C**	3818.33 ± 5.88	3547.04 ± 14.84	3044.55 ± 104.51	3144.61 ± 36.78
**Patience dock**	0.02 ± 0.01	n.d.	n.d.	n.d.
**Pollen**	175.19 ± 16.74	122.76 ± 6.20	153.67 ± 3.72	147.90 ± 2.71
**Hydrolysis collagen**	4.26 ± 0.75	1.91 ± 0.26	0.96 ± 0.04	1.06 ± 0.01
**Egg white**	4.58 ± 0.44	n.d.	0.36 ± 0.05	0.35 ± 0.02
**Sour cherries**	22.25 ± 2.73	26.81 ± 1.62	36.52 ± 0.32	29.57 ± 1.34
**Orange flavor**	2.07 ± 0.01	0.31 ± 0.04	1.31 ± 0.15	1.48 ± 0.03
**Electuary**	3.45 ± 0.26	n.d.	8.35 ± 0.73	n.d.
**The sum of the antioxidant activities related to the mass ratio of ingredients**	126.17 ± 3.28	109.36 ± 2.04	101.74 ± 3.24	102.90 ± 1.99
**Final product**	147.40 ± 1.78	139.03 ± 16.22	130.69 ± 9.66	137.52 ± 7.71
***p*** **-value**	<0.001	<0.05	<0.01	<0.01
**Synergistic effect (%)**	116.83	127.13	128.46	133.65

**Table 5:** Results for microbiological and stability indicators.

	Total Aerobic Count CFU/g	Yeast and Molds cfu/g	Water Activity Value
**NN sample 1**	<10	<10	0.345
**NN Sample 2**	<10	<10	0.404
**NN Sample 3**	<10	<10	0.353

**Table 6:** The anti-adherence activity of NN sample at different concentrations.

Strains	*Salmonella enterica* subsp. *enterica* ATCC 14028	*Escherichia coli* ATCC 25922	*Lactococcus lactis* DNS	*Enterococcus faecalis* ATCC 19433	*Lacticaseibacillus rhamnosus* ATCC 53103
**AICS** ^1^ **(%)**					
**NN**	92.12	82.03	98.03	96.44	95.57
**PBS**	97.22	99.10	100	99.17	99.01
**Positive control**	98.75	99.02	99.55	98.41	100
**AP** ^2^					
**PBS**	1	1	1	1	1
**NN**	2	2	2	2	2
**Positive control**	2	1	2	2	2

^1^ Adherence index to cellular substrate; ^2^ Adherence pattern (1—diffused; 2—diffused-aggregative; 3—aggregative).

## Data Availability

Not applicable.
